# The health advantage of volunteering is larger for older and less healthy volunteers in Europe: a mega-analysis

**DOI:** 10.1007/s10433-022-00691-5

**Published:** 2022-03-30

**Authors:** Arjen de Wit, Heng Qu, René Bekkers

**Affiliations:** 1 Department of Sociology, Center for Philanthropic Studies, Vrije Universiteit Amsterdam, Amsterdam, The Netherlands; 2 Bush School of Government and Public Service, Texas A&M University, College Station, USA

**Keywords:** Self-rated health, Volunteering, Older adults, Healthy ageing, Europe, Mega-analysis

## Abstract

There is a vast literature on the health benefits associated with volunteering for volunteers. Such health advantages are likely to vary across groups of volunteers with different characteristics. The current paper aims to examine the health advantages of volunteering for European volunteers and explore heterogeneity in the association between volunteering and health. We carry out a mega-analysis on microdata from six panel surveys, covering 952,026 observations from 267,212 respondents in 22 European countries. We provide open access to the code we developed for data harmonization. We use ordinary least squares, fixed effects, first difference, and fixed effect quantile regressions to estimate how volunteering activities and changes therein are related to self-rated health for different groups. Our results indicate a small but consistently positive association between changes in volunteering and changes in health within individuals. This association is stronger for older adults. For respondents 60 years and older, within-person changes in volunteering are significantly related to changes in self-rated health. Additionally, the health advantage of volunteering is larger for respondents in worse health. The advantage is largest at the lowest decile and gradually declines along the health distribution. The magnitude of the association at the first decile is about twice the magnitude of the association at the ninth decile. These results suggest that volunteering may be more beneficial for the health of specific groups in society. With small health advantages from year to year, volunteering may protect older and less healthy adults from health decline in the long run.

**Supplementary Information:**

The online version contains supplementary material available at 10.1007/s10433-022-00691-5.

## Introduction

Volunteering—broadly defined as providing unpaid services to others, usually through an organization—is often hailed as a positive phenomenon that not only contributes to the public good, but also to health of volunteers. It has received much attention in public debates on healthy ageing (Jenkinson et al. 2013). Plenty of research has found that volunteering is associated with better health outcomes, particularly among older adults (for reviews, see Anderson et al. 2014; Jenkinson et al. 2013). Due to the lack of data with sufficiently large sample sizes, limited studies have investigated individual differences in the health advantage across volunteers with different characteristics. The current paper examines the health advantages of volunteering for European volunteers and explores how the association between volunteering and health differs across age groups and groups with differential levels of health.

Empirical research on volunteering and health faces two challenges that we solve in the current paper. First, most empirical studies are bound to a single source of data, usually from a single country, which always poses questions around generalizability to other contexts. Several studies have overcome this problem by using data from multiple countries (e.g., Kumar et al. 2012) and document that the association between volunteering and self-rated health is present in many geographical regions of the world. None of these cross-national studies, however, was able to deal with the second challenge: the lack of harmonized longitudinal data. Longitudinal data and panel data methods are required to ascertain that the health advantage of volunteers emerges *after* they started or as they continued to volunteer. Because it is more difficult for less healthy persons to engage in volunteering, the association between health and volunteering is not only based on the benefits of volunteering but also on the health-based selection into volunteering (Li and Ferraro 2005; Papa et al. 2019). However, most longitudinal studies on the relationship between volunteering and health are based on data from a single country (Jenkinson et al. 2013), without necessarily using panel data models.

To the best of our knowledge, our paper is the first to use longitudinal data from multiple countries and apply panel data models to assess the relationship between volunteering and individual health. Our approach contributes to the literature in four ways. First, we use a new research design—mega-analysis—in which we harmonized data from six longitudinal panel surveys spanning a period of 33 years, including 952,026 observations from 267,212 respondents in 22 countries. We provide open access to the code that we developed to harmonize the data, so others can build on it in future work. Second, we take advantage of the long panel and use various panel data methods (i.e., fixed effects regressions, first difference regressions, fixed effect quantile regressions) that allow us to control for a range of time-varying factors while mitigating the influence of unobservable time-invariant factors on the relationship between health and volunteering. Third, we track the health advantage of volunteers and non-volunteers as they age, which allows for robust analyses on health decline across social groups. Fourth, we compare health advantages between healthy and less healthy persons. The results show that health advantages of volunteering are larger for Europeans who are older and/or less healthy. With robust results on individual heterogeneity in correlations between volunteering and health, our study provides additional evidence to the existing literature and contributes to public debates on health disparities and active ageing.

## Theories and hypotheses

Plenty of empirical studies have shown that volunteers report better overall health than non-volunteers among older adults. Volunteers experience lower rates of depression (Li and Ferraro 2005; Musick and Wilson 2003) and better psychological well-being (Greenfield and Marks 2004; Morrow-Howell et al. 2003; Piliavin and Siegl 2007; Thoits and Hewitt 2001) than non-volunteers. Volunteers also show slower cognitive decline (Gupta 2018; Han et al. 2020) and better cardiovascular health (Burr et al. 2016). The mortality risk among older adults who volunteer is lower than that among non-volunteers (Musick et al. 1999; Okun et al. 2013; Qu et al. 2020). Although most empirical studies have focused on older adults in one country, the health association has been found among volunteers across different age groups (e.g., adolescent volunteers: Schreier et al. 2013; midlife volunteers: Pillemer et al. 2010) and in different countries (Kumar et al. 2012).

Theories across social science disciplines provide several mechanisms that may explain the link between volunteering and health. First, physical and cognitive mechanisms—volunteering involves social, physical, and cognitive activities, which promote cardiovascular fitness and protection against cognitive decline (Anderson et al. 2014). Second, social integration theory implies that participation in voluntary associations enables the formation of new social ties (House et al. 1988). By meeting new people in voluntary associations, the size and diversity of one’s social network can be increased, providing the social support that helps overcoming health problems (Bekkers et al. 2008; Wilson 2000). A large and diverse network enables the reproduction of health-promoting social norms, sharing information, and exchanging informal care (Fiori et al. 2006; Zhang and Centola 2019). Finally, theories on psychological well-being (Ryff and Keyes 1995) imply that volunteering may have health benefits through psychological advantages. Because volunteers build up self-esteem, self-efficacy and a purpose in life by accomplishing meaningful goals, they maintain better mental health (Brown et al. 2012). Prosocial behavior also contributes to positive affect. By regulating negative emotional responses and reinforcing feelings of care and affection, giving to others buffers the effects of stress on health and well-being (Han et al. 2018; Poulin et al. 2013).

Based on the theoretical and empirical literature, we expect to find a positive association between volunteering and subjective health. Our general hypothesis is that (***H1***) changes in volunteering are positively related to changes in subjective health. We further break down changes in volunteering into trajectories, contrasting those who start volunteering with those who remain inactive; and those who continue to volunteer with those who stop volunteering. We hypothesize that (***H1a***) those who move into volunteering experience an increase in subjective health compared to those who do not volunteer and that (***H1b***) those who move out of volunteering experience a reduction in subjective health compared to those who stay in volunteering.

### Age

A few empirical studies find stronger mental health benefits associated with volunteering for the older than the younger adults (Musick and Wilson 2003; Tabassum et al. 2016; Van Willigen 2000). Research on social participation and health in social gerontology offers a number of reasons why volunteering might be more beneficial for older adults. First, gerontologists have long believed that activities promote successful ageing. According to activity theory, volunteering is beneficial for older adults as it helps them to remain active and maintain social interactions, offering channels for acquiring role supports that sustain one’s self-concept (Havighurst 1961; Herzog and House 1991).

Second, role theory further explains the benefits of volunteering in relation to the other social roles that individuals occupy. On the one hand, young adults are more likely to volunteer in response to other responsibilities and experience role strain when volunteering conflicts with other roles like being an employee, a partner, and a parent. Older adults, on the other hand, typically have a diminished number of roles and volunteering may substitute for the well-being effects of the absent roles (Chambré 1984). For example, voluntary work can partly substitute the social role of paid work among retirees (Hank and Stuck 2008). In this sense, volunteering is a more important aspect of life for older adults than it is for the younger working population.

Third, socioemotional selectivity theory poses another relevant perspective (Carstensen 1991). It suggests that older adults, perceiving limited time in the future, experience a motivational shift and deliberately choose to drop peripheral roles but maintain involvement in meaningful roles (Hendricks and Cutler 2004). While ageing, emotional motives instead of knowledge-oriented motives become more dominant, and people select the social contacts and activities which best satisfy their emotional needs. This would “select” volunteers who derive stronger psychological benefits from their volunteering. Empirical evidence shows that age moderates the relationship between social and protective motives of volunteering with well-being (Ho et al. 2012). Therefore, our hypothesis is that (***H2***) the association between volunteering and health increases with age.

### Levels of health

It is likely that less healthy individuals benefit more from volunteering because they have more to gain. Some find a stronger association between volunteering and mental health among the older volunteers who are less healthy (McDonnall 2011; Okun et al. 2011). Others find that the association between sustained volunteering and life satisfaction (and mental well-being) is stronger at the lower end of the outcome distribution than the higher end (Binder 2015; Binder and Freitag 2013). As explained by Binder and Freitag (2013: 110): “if one is already happy, frequent volunteering does not add anything, however, if one is unhappy, volunteering has a beneficial effect on one’s unhappiness.” A similar mechanism is likely to be at work for subjective health, with those at the lower end of the health distribution benefiting more from social support, psychological development, and other mechanisms that are associated with voluntary participation. In addition, research on motivations for volunteering suggests that some people volunteer to cope with their personal anxieties and negative feelings, implying that volunteering serves a protective function (Clary et al. 1998). Although it might not always be feasible to volunteer for those with functional limitations, less healthy people (particularly mental health) may actively seek to improve health by engaging in volunteer work. Hence, we expect that (***H3***) the association between volunteering and individual health is larger for those who are less healthy.

## Data, sample, and methods

### Data

This study uses a large number of observations from six longitudinal panel surveys, spanning a period of 33 years and covering 22 European countries, and pools them together in a so-called mega-analysis (Global Trust Research Consortium 2017), which in epidemiology is known as “meta-analysis of individual patient data” (Steinberg et al. 1997). The advantage of mega-analysis over meta-analysis is that it provides much more statistical power to estimate how an association varies between individuals with measured characteristics. The large number of observations also improves the external validity of the research findings and overcomes generalizability issues that are specific to a single study.

The surveys analyzed are the German Socio-Economic Panel (2018), the British Household Panel Survey and its successor Understanding Society (University of Essex, Institute for Social and Economic Research 2018), the Swiss Household Panel (2018), the Survey of Health, Ageing and Retirement in Europe (Börsch-Supan 2020; Börsch-Supan et al. 2013), the Dutch Longitudinal Internet Studies for the Social Sciences (CentERdata 2019; Scherpenzeel and Das 2010), and the Giving in the Netherlands Panel Survey (Bekkers et al. 2018). Details of each survey and descriptions of the survey instruments are provided in Online Appendix A. We harmonized responses to the questions on volunteering, subjective health, and sociodemographic characteristics of participants in these surveys to enable comparable estimates across surveys and countries. As robustness checks, we also provide results from each analysis by individual surveys in Online Appendix C. Table 1 provides an overview of the surveys that are included in this mega-analysis. At https://osf.io/zqjnb/, we provide open access to the code that we developed to harmonize the data.

**Table 1:** Surveys included in the mega-analysis

Survey	Abbreviation	Time range	Waves	Observations	Respondents
German Socio-Economic Panel	GSOEP	1984–2017	22	328,093	54,622
British Household Panel Survey/Understanding Society	BHPS/US	1996–2016	11	209,967	70,639
Swiss Household Panel	SHP	1999–2017	19	137,647	21,566
Survey of Health, Ageing and Retirement in Europe	SHARE	2004–2017	6	234,303	109,258
Dutch Longitudinal Internet Studies for the Social Sciences	LISS	2007–2017	10	34,219	8,561
Giving in the Netherlands Panel Survey	GINPS	2006–2016	6	7,797	2,566

### Dependent variable

Our dependent variable is self-rated or subjective health. Self-rated health is robustly correlated with indicators of objective health, and limitations to daily functioning and pain (Krause and Jay 1994; Mäntyselkä et al. 2003). Self-rated health is also predictive of mortality (Jylhä 2009). We obtained responses to one item on self-rated health in each survey. In the GSOEP and SHP, the question is: “How satisfied are you with your state of health?” (on a scale of 0–10). The other surveys all have a similar question on overall health (e.g., “How would you describe your health, generally speaking?”) using a 1–5 Likert scale (see Online Appendix A). We harmonized the responses by transforming them using the Percent of Maximum Scaling Possible or POMP scoring rule (Cohen et al. 1999; Moeller 2015), so that the health score ranges from 0 (worst possible health) to 100 (best possible health).

$${\text{POMP}} = [({\text{observed}} - {\text{minimum}}){/}({\text{maximum}} - {\text{minimum}})] \times 100$$

This transformation provides a metric that allows comparisons across surveys while preserving each individual’s evaluation of the health item at different time points in longitudinal data.

### Independent variable

Our key independent variable is volunteering (0: No; 1: Yes). Each survey has a survey instrument on volunteering (see Online Appendix A). Differences in wording reflect cultural differences—for example, “ehrenamtliche” (“honorary”) is a common form of volunteering in German-speaking countries. Respondents in the BHPS and GSOEP answered yes or no to a list of activities that included volunteering. Respondents in the LISS and GINPS reported if they did voluntary work for a list of organizations, a method which is likely to improve respondent’s recall of voluntary activities. In these cases, we created a dummy variable with 1 indicating volunteering in any type of organization and 0 otherwise. If the answer categories referred to volunteering intensity, we coded any voluntary activity (e.g., “At least once a week”) as 1, and no volunteering activity (e.g., “Never”) as 0.

### Covariates

We controlled for sociodemographic variables that were available in all surveys and known to be associated with volunteering and health (Musick and Wilson 2007; Papa et al. 2019). Demographic variables include age, gender (1 = *female*, 0 = *male*), and marital status (1 = *married,* 0 = *not married*). Socioeconomic variables include employment status (1 = *having a paid job,* 0 = *not working for a paid job*), retirement status *(1* = *retired; 0* = *not retired),* individual labor earnings (in Euros, Winsorized at the 99^th^ percentile and then transformed using the natural logarithm), and education attainment measured using the ISCED-97 categories, which is UNESCO’s international classification that provides comparable educational levels worldwide (0 = *Pre-primary* to 6 = *Tertiary 2nd stage*).

### Sample

The analysis sample contains 952,026 observations from 267,212 adults (18 years or older) with complete responses on all variables. For the first difference (FD) analysis (see under Analysis Plan below), the sample size is reduced because only respondents with valid observations on two consecutive waves are included. Table 2 shows descriptive statistics for the pooled data of all person-year observations.

**Table 2:** Descriptive statistics of all observations in the sample

Variable	Volunteering = 1 (*N* = 231,953)		Volunteering = 0 (*N* = 720,073)		Total (*N* = 952,026)	
	Mean	SD	Mean	SD	Mean	SD
Subjective health (0–100)	68.17	22.77	60.14	26.85	62.10	26.15
Age (18–115)	50.46	16.62	52.97	18.27	52.36	17.92
Female (0–1)	0.51	0.50	0.56	0.50	0.55	0.50
Married (0–1)	0.67	0.47	0.60	0.49	0.62	0.49
Paid job (0–1)	0.58	0.49	0.48	0.50	0.50	0.50
Retired (0–1)	0.26	0.44	0.32	0.47	0.31	0.46
Individual labor earnings (ln)	6.25	4.87	5.08	4.95	5.37	4.95
Education (ISCED-97, 0–6)	3.68	1.42	3.07	1.46	3.22	1.48

	*n*	%	*n*	%	*n*	%
0	2072	0.89	12,676	1.76	14,748	1.55
1	12,161	5.24	105,827	14.70	117,988	12.39
2	20,669	8.91	103,519	14.38	124,188	13.04
3	101,587	43.80	304,903	42.34	406,490	42.70
4	7528	3.25	21,052	2.92	28,580	3.00
5	62,539	26.96	132,761	18.44	195,300	20.51
6	25,397	10.95	39,335	5.46	64,732	6.80

Ranges (min–max) are provided in parentheses after each variable name. ln = natural logarithm

### Analysis plan

We used panel data models to test the three hypotheses. The models we apply eliminate residual confounding effects of sociodemographic variables, such as income and personality associated with volunteering and health behaviors. Our research design follows the pre-analysis plan (https://osf.io/3f6kj/).^1^ Our main estimates of the longitudinal relationship between changes in volunteering and changes in health come from three types of regression models. In all models, we use a two-sided test with a 5% alpha to test whether the null hypothesis can be rejected.

First, we examine the relationship between health and volunteering using fixed effects (FE) models, controlling for individual and time fixed effects and various covariates. To illustrate the importance of model specification, we contrast these estimates with the cross-sectional estimate from an ordinary least squares (OLS) model without fixed effects. Standard errors are clustered by respondent. Results are expressed in health score (POMP) on the scale of 0 (worst possible health) to 100 (best possible health).

Second, we predict the change in health with first difference (FD) models. Here, the dependent variable is the change in self-rated health from the previous survey wave (*t* − 1) to the current (*t*). Note that the time range of the change varies per survey, with spells between waves varying between 1 year (e.g., SHP) and 4 years (SHARE). We run two separate sets of analyses, (1) on a subset of respondents who volunteered at *t* − 1, in order to compare continuous volunteers with volunteers who quit, and (2) on a subset of respondents who did not volunteer at *t* − 1, in order to compare non-volunteers who join volunteering with continuous non-volunteers. Again, standard errors are clustered by individual respondents.

The results of the first two regression models are a test of Hypotheses 1 and 2. For H2, we examine the volunteering–health association for different age groups separately. By comparing the confidence intervals, we see whether the split-sample coefficients significantly differ from zero, and from each other. To test H3, we use a third regression approach. Here, we use fixed effects quantile regressions to examine whether the association between volunteering and subjective health varies along the health distribution. Different from the least square methods that estimate the conditional mean, quantile regressions estimate the conditional quantiles of the dependent variable. We used the new approach developed by Machado and Santos Silva (2019: 145). The regressions control for the full set of covariates, individual fixed effects, and time dummies.

## Results

### Descriptive statistics: volunteers have a 13% health advantage

**Fig. 1:**
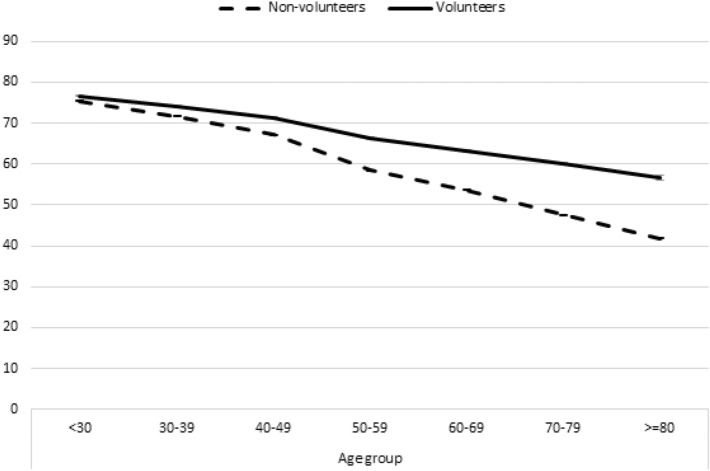
Average self-rated health among volunteers and non-volunteers by age group. *Note*: Cross-sectional averages, not controlled for covariates

In the pooled sample, volunteers on average report a health score of 68, which is 8 points higher than the score of 60 for non-volunteers. Taking the score for non-volunteers as a baseline, volunteers on average have a health advantage of more than 13%. The health advantage of volunteers increases strongly with age (Fig. 1). Among respondents younger than 40, we see no health advantage for volunteers. Among respondents in their seventies, the health advantage of volunteers is 26%; among respondents 80 years and older, it is 35%.

### H1: small association between changes in volunteering and changes in health

**Fig. 2:**
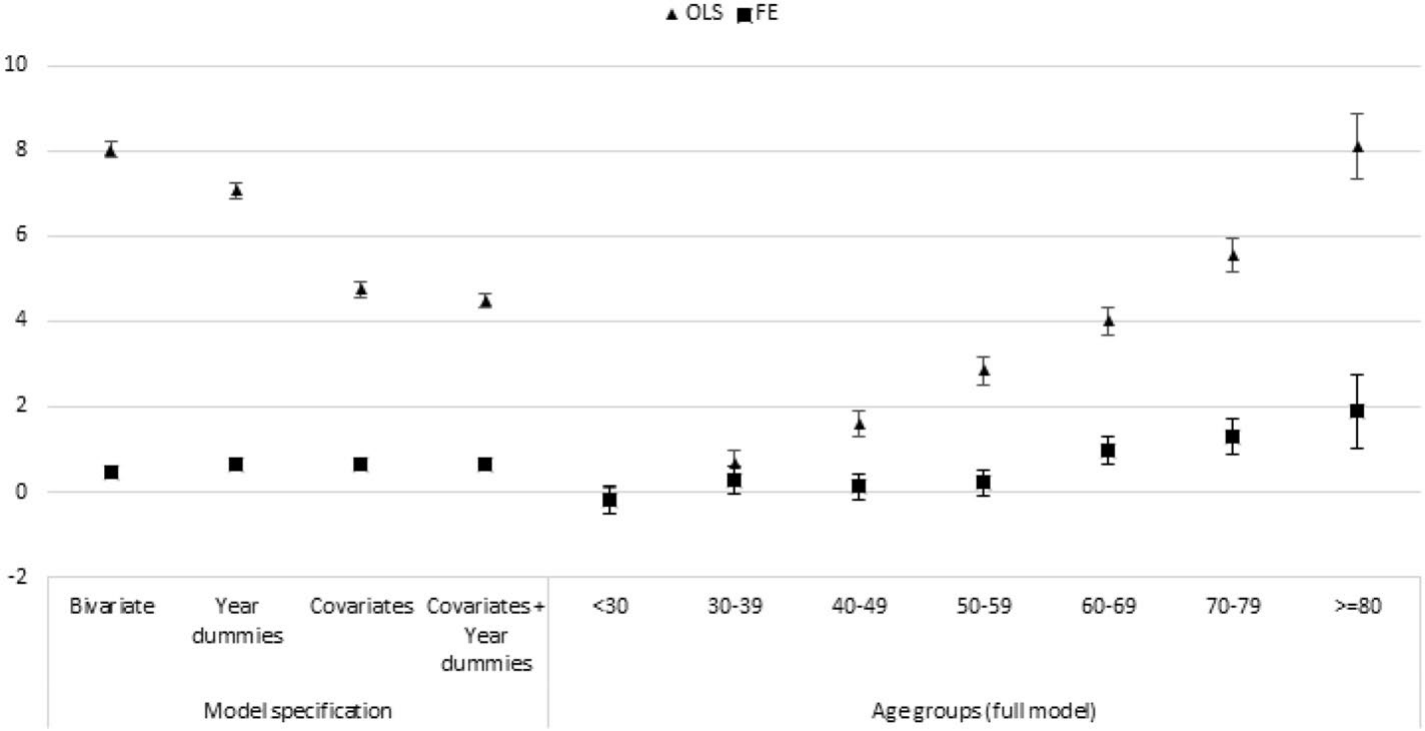
OLS and fixed effects regression coefficients of volunteering on self-rated health by model specification and age group. *Note*: Estimates derived from different regression models without control variables (Bivariate); including dummies for survey year (Year dummies); controlled for female, age, married, paid job, retired, labor earnings (ln); and educational level in ISCED-97 levels (Covariates); and both dummies for survey year and control variables (Covariates + Year dummies). Estimates by age groups are derived from full models with control variables and year dummies. All models are estimates with (FE) and without (OLS) fixed effects for individual respondents

In the bivariate OLS regression using the pooled dataset, the coefficient of volunteering is 8.03 (*p* < 0.01), and it becomes 4.47 (*p* < 0.01) when including year dummies and individual-level covariates. When adding individual fixed effects, the coefficient of volunteering drops to 0.45 (*p* < 0.01) in the bivariate regression and 0.66 (*p* < 0.01) in the model with full controls. The fact that the FE coefficients are much smaller than the OLS coefficients suggests that the health advantage of volunteers is primarily due to pre-existing differences between volunteers and non-volunteers. Still, the positive FE coefficient indicates that volunteers have a small health advantage, even when accounting for their pre-existing differences with non-volunteers. Figure 2 shows the coefficients of volunteering in different OLS regression models on all person-year observations (the triangles in the figure) and models that include individual fixed effects (the squares in the figure). Tables 4 and 5 in Online Appendix provide full results of various model specifications.

### H1a and H1b: largest health advantage for continuous volunteers

The FD models confirm that there is a small, but statistically significant health advantage of volunteering. *Joining volunteering* is associated with a 0.48-point (*p* < 0.01) increase in self-rated health in a bivariate analysis. Controlling for covariates as well as year, survey, and country effects, the coefficient of joining volunteering decreases to 0.43 (*p* < 0.01). The coefficient of *continuous volunteering* is larger than that of quitting volunteering; continuous volunteers experience a 0.6-point (*p* < 0.01) health advantage in the full model that controls for individual covariates as well as year, survey, and country dummies.

**Fig. 3:**
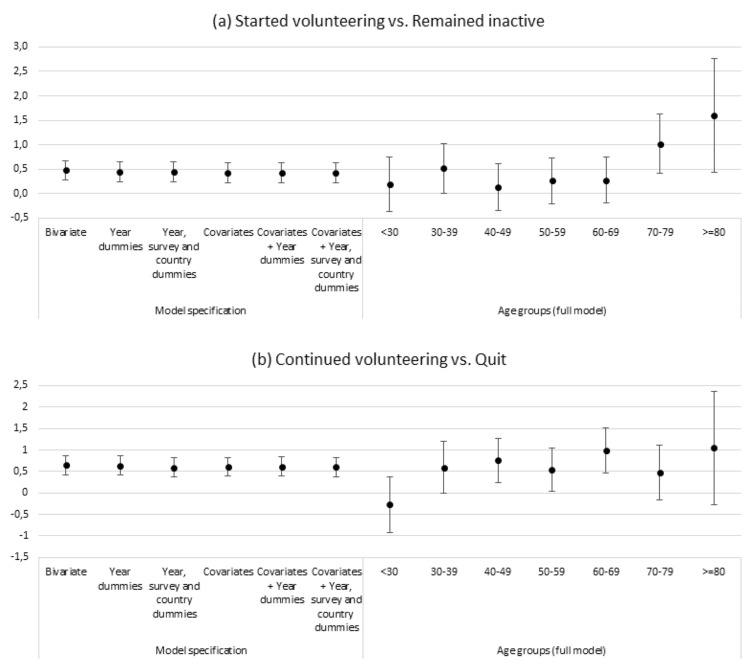
Coefficients from first-difference regression models of Δ volunteering on Δ self-rated health. *Note*: Estimates derived from different regression models: without control variables (Bivariate); including dummies for survey year (Year dummies); including dummies for survey year, dummies for survey and dummies for country of residence (Year, survey, and country dummies); controlled for female, age, married, paid job, retired, labor earnings (ln) and educational level in ISCED-97 levels (Covariates); both control variables and dummies for survey year (Covariates + Year dummies); and control variables, dummies for survey year, dummies for survey, and dummies for country of residence (Covariates + Year, survey, and country dummies). Estimates by age groups are derived from full models with control variables and year, survey, and country dummies

The left-hand part of Fig. 3a, b shows the main results of the first-difference analyses in different model specifications. Complete regression results are provided in Online Appendix, Table 6.

### H2: the health advantage of volunteers is larger among older adults

In both the FE and the FD analyses, we estimated models for different age groups separately. The results show that associations are more strongly positive among older adults. As shown in the right-hand panel of Fig. 2, the FE coefficient of volunteering is close to zero and not significant for respondents in their tens, twenties, thirties, forties, and fifties. Only for respondents 60 years and older, within-person changes in volunteering are significantly related to changes in self-rated health. For respondents 80 years and older, the FE coefficient is 1.89.

The right-hand panel of Fig. 3a, b represents the results from the model with full controls across different age groups. Regarding starting volunteering (vs. not volunteering), all coefficients are positive, but only among respondents 70 years and older the volunteering coefficient is statistically significant. Comparing continuous volunteers with volunteers who quit (Fig. 3b), the coefficient is below zero and not significant among respondents younger than 30 years. In the older age groups, the coefficient is positive ranging from 0.5 to 1. For the age groups between 40 and 69, the coefficients are statistically significant.

### H3: the health advantage of volunteering is larger for those in worse health

**Fig. 4:**
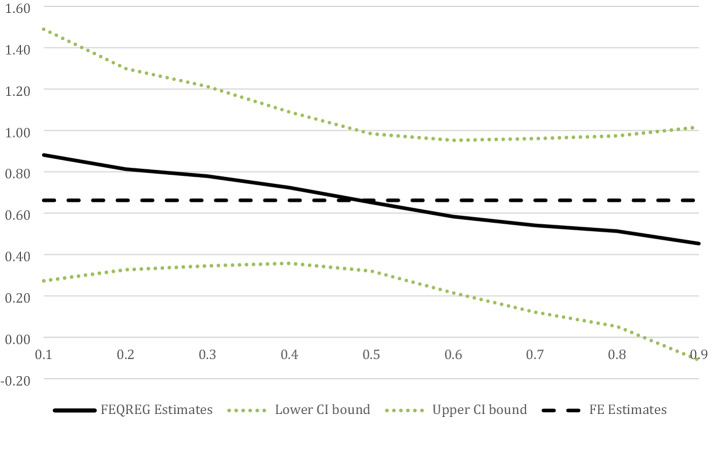
Health advantage of volunteering by decile of health, fixed effects quantile regression results. *Note*: Estimates derived from regression models including fixed effects for individual respondents and controlled for age, married, paid job, retired, labor earnings (ln), and educational level in ISCED-97 levels

The fixed effects quantile regression results show that the association between volunteering and health is positive and significant across deciles of health, except for the top decile where the positive association becomes non-significant. Moreover, the association is the strongest at the lowest decile and gradually declines along the health distribution. The magnitude of the association at the first decile (*β* = 0.88, *p* = 0.005) is about twice the magnitude of the association at the ninth decile (*β* = 0.45, *p* = 0.115). The results provide support for the stronger association between volunteering and health among those in poorer health. Figure 4 displays the results, with the full regression table in Online Appendix Table 8.

In split-sample results by age group, for the group of participants who were 65 years or older, the association between volunteering and health was positive and statistically significant across all deciles of health, with the association declining from the lowest (*β* = 1.86, *p* < 0.0001) to the top decile (*β* = 1.11, *p* < 0.0001). In contrast, the association was smaller at every decile for respondents younger than 65 (0.40 to 0.16), and it was only statistically significant from the second to fourth decile. These results provide additional evidence that the health advantage of volunteering is larger for older adults at different levels of health.

## Discussion and conclusion

### Discussion

The current study, utilizing a large longitudinal dataset and various panel data methods, shows how the association between volunteering and individual health differs across age groups and groups with differential levels of health. We find evidence that supports all three hypotheses. First, we find that there is a small, but statistically significant health advantage of volunteering, providing support for H1. This is in line with previous empirical studies that find volunteers report better self-rated health than non-volunteers (e.g., Kumar et al. 2012; Piliavin and Siegl 2007). More importantly, we add to the literature by showing that respondents who started volunteering have a health advantage over those who stayed inactive, and that respondents who continue to volunteer have a health advantage to those who quit volunteering.

Second, across analyses, we consistently find that the association between volunteering and health increases with age, supporting H2. The FE regressions show that only for respondents 60 years and older, within-person changes in volunteering are significantly related to changes in self-rated health. In the FD regressions, joining volunteering is significantly associated with an increase in self-rated health only among respondents 70 years and older, and continuing to volunteer is statistically significant among respondents between 40 and 69 years old. These findings provide additional evidence to the limited studies that find stronger benefits associated with volunteering among the older than the younger adults (Musick and Wilson 2003; Tabassum et al. 2016; Van Willigen 2000).

Third, consistent with H3, the results from fixed effects quantile regressions find that the association between volunteering and health decreases in magnitude along the health distribution, with the association being larger for those with poorer health and smaller for those who have better health. Previous research has shown stronger mental health benefits associated with volunteering among less healthy older volunteers (McDonnall 2011; Okun et al. 2011), and a stronger association between volunteering and life satisfaction for volunteers who are less happy to begin with (Binder 2015; Binder and Freitag 2013). Our study is among the first to provide support for a heterogeneous association between volunteering and self-rated health.

### Strengths, limitations, and future research

The current mega-analysis delivers a substantial contribution to the literature by providing robust evidence on how the advantages of volunteering differ between groups of volunteers. We show results across a large number of European countries, checking for the robustness of the results. There is quite some variance in the coefficients from different countries. It is likely that findings diverge due to political, economic, social, and cultural differences between countries. Moreover, it is important to take differences in survey methodologies and data quality into account. Our main analyses used panel data methods, accounting for the country and survey fixed effects that may affect the association between volunteering and health. Notably, for the countries with the most precise estimates—Germany, Switzerland, and the UK—the coefficients are not far from each other, and all close to the average estimate. This pattern suggests that with more observations we come closer to the “true” association. Our estimates are consistent with those obtained in meta-analyses on volunteering and health (Anderson et al. 2014; Jenkinson et al. 2013), indicating no or positive associations between volunteering and different health measures like functional abilities, depressive symptoms, and mortality. We show that a mega-analysis is a good alternative for a meta-analysis, because it allows for even more precise estimates and for robust subgroup analyses.

Admittedly, this study has its limitations, which also offer pathways for future research. First, as in other research relying on survey data, we caution that our estimates provide insight only among those who are relatively healthy, at least healthy enough to participate in the survey. Longitudinal survey participants are above average in health (Golomb et al. 2012), reducing the variance in health. Particularly in surveys among older people, the dropout of the panel is health related and not observed in the data we analyze. Such selective panel attrition implies that declines in health are underestimated, and these declines are particularly likely for non-volunteers and those who stopped volunteering. Thus, our estimates are lower bound estimates of changes that are likely to be larger in the population.

Second, a recurring limitation in this body of the literature is causal inference. We stress that causality cannot be inferred from our results. Providing volunteer opportunities in field experiments (Jiang et al. 2021; Pettigrew et al. 2015, 2019) is one of the best ways to assess the advantages of volunteering, but this is not always practically and morally possible. The data we compiled provide two promising opportunities to address causal inference in future research. First, the data cover a long period in which natural experiments occurred, such as natural disasters and changes in the mandatory school age due to compulsory schooling laws, which create exogenous shocks that increase volunteering. Second, the data include measures that may be used as instrumental variables. Functional limitations of the spouse, for example, may be correlated with changes in volunteering but not with their consequences in terms of health (Gupta 2018).

Third, there may be biases or measurement issues. The survey question on volunteering in the past 12 months is sensitive to recall bias. There might also be social desirability bias if either volunteers or non-volunteers tend to report more positively on their health. If these biases are non-random across levels self-rated health, this would affect the precision of the estimates. Furthermore, some surveys have more than 1 year between the waves, so we do not observe changes in volunteering or health in between the observations.

Fourth, the current study provides robust evidence supporting the differential associations between volunteering and health based on comprehensive analyses of a large dataset; however, there are other important questions to be examined. For example, it does not directly test the potential mechanisms producing the associations or distinguish between different types of voluntary activities that may produce differential outcomes (e.g., McMunn et al. 2009; Wahrendorf et al. 2008). In addition, we did not examine the hours volunteered (Morrow-Howell et al. 2003): Because not every survey we harmonized provides information on the frequency of volunteering, we began by focusing on the dichotomous variable of volunteering to obtain the largest sample size possible. Future research may elect to use a smaller subset of the dataset to examine these questions. With its long-time frame and large number of participants, the current dataset is very well suited for extensive analyses on health decline and other trajectories. Moreover, the data include other indicators of well-being and health like physical limitations, specific diseases, mental health, and life satisfaction.

### Conclusion

Our mega-analysis of six large longitudinal surveys from Europe provides robust evidence on the association between volunteering and individual health. Volunteering is not a panacea for health. Much of the association is due to self-selection of healthier individuals into volunteering and the selection of less healthy individuals out of volunteering. Nonetheless, taking such selection processes into account, we still find that health improves when Europeans start volunteering and declines when they stop volunteering. Even though the annual advantage is small, it is important because it accumulates over time. The health advantage of volunteering for volunteers is similar in magnitude to the health disadvantage of ageing 1 year. We also find considerable heterogeneity in these changes. The health advantage increases with age, with the most substantial advantages for volunteers aged 60 and over. The health advantage of volunteering among those in worse health is twice as large as the health advantage among the healthiest Europeans.

The findings of this study are relevant for public policy. If volunteering can prevent health decline among older adults and the least healthy, as our results suggest, it could reduce costs of medical treatments. For volunteers and voluntary organizations, this is a stimulus to continue their efforts. Our findings suggest that volunteering is not only a form of social engagement with positive consequences for society as a whole, but may also have positive health benefits for individuals, particularly for the more vulnerable.

## Supplementary Information

Below is the link to the electronic supplementary material.

Supplementary file1 (DOCX 116 kb)

## Data Availability

Data from the different surveys used in this paper are available upon request at the responsible research institutes.

## Footnotes

1 For this paper, we analyzed Models 1, 2, 4, 5.1, 6, and 7 of the pre-analysis plan. To keep the paper within space limits, some of the pre-registered models are in the online appendices rather than the main text. Online Appendix D includes a description of dynamic panel data (DPD) models. Our exploratory analysis did not find valid instruments for the DPD model, and we concluded that DPD was not an appropriate approach for our question.

## Code availability

The code that is used to merge, recode, and analyze the data is publicly available at https://osf.io/zqjnb/.
