# Supplementary material for: The health advantage of volunteering is larger for older and less healthy volunteers in Europe: a mega-analysis
Source: Eur J Ageing. 2022 Mar 30;19(4):1189–200. doi: 10.1007/s10433-022-00691-5 (PMC9729491; doi:10.1007/s10433-022-00691-5)
Supplement: Supplementary file 1 — Supplementary file1 (DOCX 116 kb) [file 10433_2022_691_MOESM1_ESM.docx]

## APPENDIX A: SURVEYS AND SURVEY INSTRUMENTS

**Surveys**

The *German Socio-Economic Panel (GSOEP)* (Socio-Economic Panel, 2018) is Europe’s longest running household panel survey, including questions on a wide range of socio-economic issues. The survey started in 1984 with a nationally representative sample of households in Germany. In 1990 households from Eastern Germany were added to the sample. A sample of immigrants was added in 1994/95 and a sample of wealthy households in 2002. The survey is conducted face-to-face; from 1994/95 onwards computers are used (CAPI).

The SOEP data are available through DIW Berlin. For more information and accessing the data see<https://www.diw.de/en/diw_02.c.222518.en/research_data_center_of_the_soep.html>.

The *British Household Panel Survey (BHPS)* (University of Essex, Institute for Social and Economic Research, 2018) is a long-running panel with questions on a wide range of socio-economic issues. A nationally representative sample is drawn of households in Great Britain, Wales, Scotland and Northern Ireland, whose members are interviewed at their home if possible. At Wave 9 of the BHPS, the survey moved from a pen-and paper (PAPI) mode of data collection to Computer Assisted Personal Interview (CAPI). In 2009 the BHPS was succeeded by *Understanding Society*, for which the same panel of people was asked to join the new survey.

The BHPS and US data are available through the UK Data Service. For more information and accessing the data see<https://www.understandingsociety.ac.uk/documentation/access-data>

The *Swiss Household Panel (SHP)* (Swiss Household Panel, 2018) is an annual survey among a random group of households in Switzerland since 1999. The survey includes questions on a wide range of social and economic issues. Data is collected through Computer Assisted Telephone Interviews (CATI).

The SHP data are available through FORSbase. For more information and accessing the data see<https://forscenter.ch/projects/swiss-household-panel/data/>

The *Giving in the Netherlands Panel Survey (GINPS)* (Bekkers et al., 2018) is a biennial survey among a representative sample of Dutch households which includes extensive modules on charitable giving, prosocial values, volunteering and informal helping. The first wave of the panel survey took place in 2002. Respondents are in a database of people who agreed to participate in a survey every once in a while and are interviewed online (CAWI).

The GINPS data are available through OSF. For more information and accessing the data see <https://osf.io/3s2fh/>

The *Longitudinal Internet studies for the Social Sciences (LISS)* panel (CentERdata, 2019; Scherpenzeel & Das, 2010) is an annual survey which includes a number of recurring core modules as well as incidental modules on specific topics in collaboration with different social researchers. The panel is based on a true probability sample of households drawn from the population register by Statistics Netherlands. Data is collected online (CAWI). Households that could not otherwise participate are provided with a computer and Internet connection.

The LISS panel data are available through CentERdata. For more information and accessing the data see<https://www.lissdata.nl/access-data>

The *Survey of Health, Ageing and Retirement in Europe (SHARE)* is a large cross-national panel among people aged 50 years or older. Some countries joined the survey later than others. Data is mainly collected through Computer Assisted Personal Interviewing (CAPI). Wave 3, which is a special wave on life events, is not used for this study.

For more information and accessing the data see<http://www.share-project.org/data-access.html>

**Survey instruments**

*Self-rated health* measures are included in all surveys, typically with response categories on 5 points Likert scales. The GSOEP includes current health status only since 1992, while satisfaction with health (0=Completely dissatisfied, 10=Completely satisfied) goes back to the first wave of the survey. We choose to use the latter in order to maintain the largest number of observations. A similar measure is available in the SHP. Understanding Society uses a different question with different response categories compared with the BHPS. The question in the BHPS is the only one that explicitly mentions a target period, asking respondents to think back over the last 12 months. The first SHARE questionnaire used two different categorizations and randomly assigned respondents to one of them. In order to have similar categories we only included respondents who were given the scaling that continued throughout other waves.

*Volunteering* is measured in different ways across the available datasets. In most surveys, only one question on volunteering is included, without an explanation of the term volunteering. This type of question is likely to yield an underestimate of the total number of volunteer activities when respondents do not recognize their activities as volunteering and because people may forget episodic volunteering activities. Respondents in the BHPS go through a list of possible leisure activities in which ‘unpaid voluntary work’ is included. This measure is similar to the question in the GSOEP how frequently respondents perform volunteer work (‘ehrenamtliche tätigkeiten’). The SHP explicitly mentions ‘honorary or voluntary activities within an association, an organisation or an institution’, where ‘honorary’ is a translation of ‘ehrenamtliche’, which is a common form of volunteering in German-speaking countries. Both the GINPS and the LISS provide respondents with a list of possible organisations people can be involved in, which is likely to raise the number of people who indicate that they volunteer (Rooney, Steinberg & Schervish, 2004). The GINPS, LISS and Understanding Society mention a reference period of the past 12 months. The SHARE changed the reference period from the last month (wave 1 and 2) to the past twelve months (wave 4 onwards).

The different survey instruments on volunteering refer to different time frames. In the Understanding Society, GINPS, LISS, and waves 4-7 of the SHARE, respondents reported if they volunteered in the past 12 months. In waves 1-2 of the SHARE, the time frame is ‘the last month’. The survey instruments in the GSOEP, BHPS and SHP did not mention a reference period.

The reference periods of the survey instruments may affect the estimated *association between volunteering and health*. When no time frame is specified, respondents are likely to think about current health or current voluntary activities. When respondents are explicitly asked to think back over the last 12 months, they are more likely to recall events in the (recent) past. The formulation of the questions may thus affect the extent to which empirical estimates capture the causal relation from volunteering to health, or reverse causation. When volunteering refers to the past year and the health question does not have a specific time period – which is the case in Understanding Society, the GINPS, LISS, and waves 4-7 of the SHARE – the chronology is in line with the hypothesized pathway from volunteering to health. When both instruments refer to the past 12 months, or both instruments do not specify a time frame, the estimations probably capture both the volunteering-health association and the reverse pathway from health to volunteering.

***Table 3*** *Wording of survey instruments on volunteering and self-rated health*

|  | **Volunteering** | **Self-rated health** |
| --- | --- | --- |
| GSOEP | Which of the following activities do you take part in during your free time?  […]  - Volunteer work in clubs or social services  *1 At least once a week (1)*  *2 At least once a month (1)*  *3 Less often (1)*  *4 Never (0)* | How satisfied are you with your health?  *0 Completely dissatisfied (0)*  *1 (1)*  *2 (2)*  *3 (3)*  *4 (4)*  *5 (5)*  *6 (6)*  *7 (7)*  *8 (8)*  *9 (9)*  *10 Completely satisfied (10)* |
| BHPS | For each of the following leisure activities, please tick the box to show how often you do each of these things.  […]  - Do unpaid voluntary work  *1 At least once a week (1)*  *2 At least once a month (1)*  *3 Several times a year (1)*  *4 Once a year or less (1)*  *5 Never almost never (0)* | Please think back over the last 12 months about how your health has been. Compared to people of your own age, would you say that your health has on the whole been ...  *1 Excellent (4)*  *2 Good (3)*  *3 Fair (2)*  *4 Poor (1) or*  *5 Very Poor (0)?* |
| US | In the last 12 months, have you given any unpaid help or worked as a volunteer for any type of local, national or international organisation or charity?  *1 Yes (1)*  *2 No (0)* | In general, would you say your health is...  *1 Excellent (4)*  *2 Very good (3)*  *3 Good (2)*  *4 Fair (1)*  *5 Poor (0)* |
| SHP | Do you have honorary or voluntary activities within an association, an organisation or an institution?  Voluntary activities relating to private initiative, such as helping neighbours, at local fetes are not included here; payments for meetings, expenses or payment of symbolic amounts are not considered as forms of  remuneration.  *1 Yes (1)*  *2 No (0)* | How satisfied are you with your state of health, if 0 means "not at all satisfied" and 10 "completely satisfied"?  *0 Not at all satisfied (0)*  *1 (1)*  *2 (2)*  *3 (3)*  *4 (4)*  *5 (5)*  *6 (6)*  *7 (7)*  *8 (8)*  *9 (9)*  *10 Completely satisfied (10)* |
| GINPS | Now the question is whether you are a volunteer for an organization. With volunteer work, we mean tasks you do not receive a salary for, but possibly an expense allowance. In the past 12 months, have you been a volunteer at an organization on the following fields:  - Sports  - Health care  - Social work, legal assistance, probation and victim service  - Education: schools, adult education  - Culture and arts  - Community work  - Neighbourhood association and interest group, housing/ tenants association  - Environmental protection  - Nature conservation  - Animal welfare  - Politics  - Trade Union, professional organization  - Refugee assistance, human rights  - Religion  - Organization for ethnic minorities  - Recreation, hobby  - Developmental aid  - Other  *0 No (0)*  *1 Yes (1 if any)* | What do you think about your health in general?  *1 Excellent (4)*  *2 Very good (3)*  *3 Good (2)*  *4 Poor (1)*  *5 Bad (0)* |
| LISS | We now list a number of organizations that you are free to join. Can you indicate, for each of the organizations listed, what applies to you at this moment or has applied to you over the past 12 months?  - a sports club or club for outdoor activities  - a cultural association or hobby club  - a trade union  - a business, professional or agrarian organization  - a consumers’ organization or automobile club  - an organization for humanitarian aid, human rights, minorities or migrants  - an organization for environmental protection, peace organization or animal rights organization  - a religious or church organization  - a political party  - a science, education, teachers’ or parents’ association  - a social society; an association for youth, pensioners/senior citizens, women; or friends’ clubs  - other organizations that you can freely join  *1 no connection (0)*  *2 donated money (0)*  *3 participated in an activity (0)*  *4 member (0)*  *5 performed voluntary work (1 if any)* | How would you describe your health, generally speaking?  *1 Poor (0)*  *2 Moderate (1)*  *3 Good (2)*  *4 Very good (3)*  *5 Excellent (4)* |
| SHARE | Have you done any of these activities in the last month? (from wave 4 onwards: which of the activities listed on this card - if any - have you done in the past twelve months?)  - Done voluntary or charity work  *(0 if not selected)*  *(1 if selected)* | Would you say your health is...  *1 Excellent (4)*  *2 Very good (3)*  *3 Good (2)*  *4 Fair (1)*  *5 Poor (0)* |

## APPENDIX B: REGRESSION TABLES

***Table 4*** *OLS regression models of self-rated health*

|  | Bivariate | Year FE | Covariates | Covariates + Year FE | Covariates + Year FE + Survey and country FE |
| --- | --- | --- | --- | --- | --- |
|  |  |  |  |  |  |
| Volunteer | 8.032*** | 7.074*** | 4.742*** | 4.470*** | 2.808*** |
|  | (0.0929) | (0.0913) | (0.0887) | (0.0874) | (0.0813) |
| Female |  |  | 0.328*** | 0.367*** | -0.105 |
|  |  |  | (0.0960) | (0.0940) | (0.0873) |
| Age |  |  | -0.419*** | -0.368*** | -0.334*** |
|  |  |  | (0.00339) | (0.00349) | (0.00336) |
| Married |  |  | 0.944*** | 0.889*** | 1.415*** |
|  |  |  | (0.0953) | (0.0932) | (0.0859) |
| Paid job |  |  | 2.356*** | 2.747*** | 4.498*** |
|  |  |  | (0.114) | (0.113) | (0.109) |
| Retired |  |  | 3.526*** | 3.448*** | 3.714*** |
|  |  |  | (0.146) | (0.143) | (0.137) |
| Labor earnings (ln) |  |  | 0.772*** | 0.695*** | 0.295*** |
|  |  |  | (0.0121) | (0.0120) | (0.0116) |
| Reference: ISCED-97: 0 |  |  |  |  |  |
| ISCED-97: 1 |  |  | 1.792*** | 0.924*** | -1.811*** |
|  |  |  | (0.299) | (0.295) | (0.291) |
| ISCED-97: 2 |  |  | 5.263*** | 4.159*** | 2.124*** |
|  |  |  | (0.303) | (0.297) | (0.292) |
| ISCED-97: 3 |  |  | 9.525*** | 8.028*** | 3.832*** |
|  |  |  | (0.287) | (0.282) | (0.279) |
| ISCED-97: 4 |  |  | 7.121*** | 6.512*** | 5.095*** |
|  |  |  | (0.369) | (0.362) | (0.351) |
| ISCED-97: 5 |  |  | 11.07*** | 10.47*** | 6.265*** |
|  |  |  | (0.296) | (0.292) | (0.291) |
| ISCED-97: 6 |  |  | 11.98*** | 9.618*** | 6.463*** |
|  |  |  | (0.335) | (0.331) | (0.331) |
| Individual FE | NO | NO | NO | NO | NO |
| Year FE | NO | YES | NO | YES | YES |
| Survey FE | NO | NO | NO | NO | YES |
| Country FE | NO | NO | NO | NO | YES |
| Constant | 60.14*** | 67.28*** | 67.44*** | 70.26*** | 82.90*** |
|  | (0.0589) | (0.285) | (0.326) | (0.405) | (0.557) |
| R-squared | 0.017 | 0.084 | 0.191 | 0.210 | 0.280 |

N=952,026. Robust standard errors in parentheses. *** p<0.01, ** p<0.05, * p<0.1

***Table 5*** *OLS regression models of self-rated health, with fixed effects for individuals*

|  | Bivariate | Year FE | Covariates | Covariates + Year FE |
| --- | --- | --- | --- | --- |
|  |  |  |  |  |
| Volunteer | 0.450*** | 0.666*** | 0.636*** | 0.662*** |
|  | (0.0651) | (0.0630) | (0.0630) | (0.0629) |
| Age |  |  | -0.591*** | -0.970 |
|  |  |  | (0.00615) | (0.706) |
| Married |  |  | 0.249** | 0.224** |
|  |  |  | (0.108) | (0.109) |
| Paid job |  |  | 1.356*** | 1.356*** |
|  |  |  | (0.0939) | (0.0939) |
| Retired |  |  | 1.946*** | 2.007*** |
|  |  |  | (0.122) | (0.122) |
| Labor earnings (ln) |  |  | 0.0354*** | 0.0365*** |
|  |  |  | (0.0102) | (0.0101) |
| Reference: ISCED-97: 0 |  |  |  |  |
| ISCED-97: 1 |  |  | -2.553*** | -2.681*** |
|  |  |  | (0.461) | (0.462) |
| ISCED-97: 2 |  |  | -0.606 | -0.653 |
|  |  |  | (0.408) | (0.407) |
| ISCED-97: 3 |  |  | -0.427 | -0.391 |
|  |  |  | (0.360) | (0.359) |
| ISCED-97: 4 |  |  | 0.0447 | 0.136 |
|  |  |  | (0.490) | (0.489) |
| ISCED-97: 5 |  |  | 0.184 | 0.141 |
|  |  |  | (0.408) | (0.407) |
| ISCED-97: 6 |  |  | 0.179 | 0.271 |
|  |  |  | (0.456) | (0.455) |
| Individual FE | YES | YES | YES | YES |
| Year FE | NO | YES | NO | YES |
| Constant | 61.99*** | 76.96*** | 91.81*** | 104.8*** |
|  | (0.0159) | (0.285) | (0.456) | (20.66) |
| R-squared | 0.000 | 0.034 | 0.033 | 0.035 |

N=952,026 (267,212 persons). Robust standard errors in parentheses. *** p<0.01, ** p<0.05, * p<0.1

***Table 6*** *First-difference regression models of Δ self-rated health, among respondents who did not volunteer at t-1*

|  | I | II | III | IV | V | VI |
| --- | --- | --- | --- | --- | --- | --- |
|  |  |  |  |  |  |  |
| Volunteer_t_ | 0.475*** | 0.448*** | 0.448*** | 0.420*** | 0.415*** | 0.430*** |
|  | (0.104) | (0.104) | (0.104) | (0.105) | (0.105) | (0.105) |
| Female |  |  |  | 0.292*** | 0.278*** | 0.271*** |
|  |  |  |  | (0.0510) | (0.0510) | (0.0512) |
| Age |  |  |  | -0.0294*** | -0.0270*** | -0.0250*** |
|  |  |  |  | (0.00220) | (0.00229) | (0.00230) |
| Married |  |  |  | -0.0607 | -0.0443 | -0.0380 |
|  |  |  |  | (0.0541) | (0.0538) | (0.0539) |
| Paid job |  |  |  | 0.426*** | 0.408*** | 0.496*** |
|  |  |  |  | (0.133) | (0.133) | (0.135) |
| Retired |  |  |  | 0.451*** | 0.439*** | 0.519*** |
|  |  |  |  | (0.104) | (0.104) | (0.106) |
| Labor earnings (ln) |  |  |  | -0.0254* | -0.0267** | -0.0357*** |
|  |  |  |  | (0.0131) | (0.0131) | (0.0135) |
| ISCED-97: 0 |  |  |  | ref. | ref. | ref. |
| ISCED-97: 1 |  |  |  | -0.807*** | -0.945*** | -0.625** |
|  |  |  |  | (0.286) | (0.288) | (0.298) |
| ISCED-97: 2 |  |  |  | -0.620** | -0.697** | -0.506* |
|  |  |  |  | (0.287) | (0.287) | (0.301) |
| ISCED-97: 3 |  |  |  | -0.727*** | -0.862*** | -0.675** |
|  |  |  |  | (0.282) | (0.283) | (0.297) |
| ISCED-97: 4 |  |  |  | -1.319*** | -1.379*** | -1.048*** |
|  |  |  |  | (0.326) | (0.326) | (0.342) |
| ISCED-97: 5 |  |  |  | -0.851*** | -0.978*** | -0.728** |
|  |  |  |  | (0.285) | (0.286) | (0.300) |
| ISCED-97: 6 |  |  |  | -0.570* | -0.677** | -0.507 |
|  |  |  |  | (0.301) | (0.303) | (0.316) |
| Year FE | NO | YES | YES | NO | YES | YES |
| Survey FE | NO | NO | YES | NO | NO | YES |
| Country FE | NO | NO | YES | NO | NO | YES |
| Constant | -1.089*** | -1.181*** | -1.940*** | 0.904*** | 0.573 | -0.547 |
|  | (0.0256) | (0.275) | (0.427) | (0.304) | (0.406) | (0.527) |
| R-squared | 0.000 | 0.002 | 0.003 | 0.001 | 0.002 | 0.003 |

N=379,706 (147,663 persons). Robust standard errors in parentheses. *** p<0.01, ** p<0.05, * p<0.1

***Table 7*** *Results from first-difference regression models of Δ self-rated health, among respondents who volunteered at t-1*

|  | I | II | III | IV | V | VI |
| --- | --- | --- | --- | --- | --- | --- |
|  |  |  |  |  |  |  |
| Volunteer_t_ | 0.648*** | 0.640*** | 0.589*** | 0.609*** | 0.610*** | 0.600*** |
|  | (0.113) | (0.113) | (0.114) | (0.113) | (0.114) | (0.115) |
| Female |  |  |  | 0.159* | 0.139 | 0.173** |
|  |  |  |  | (0.0870) | (0.0874) | (0.0877) |
| Age |  |  |  | -0.0212*** | -0.0217*** | -0.0187*** |
|  |  |  |  | (0.00397) | (0.00407) | (0.00407) |
| Married |  |  |  | 0.262*** | 0.279*** | 0.279*** |
|  |  |  |  | (0.0974) | (0.0979) | (0.0978) |
| Paid job |  |  |  | 0.280 | 0.292 | 0.345 |
|  |  |  |  | (0.210) | (0.210) | (0.212) |
| Retired |  |  |  | -0.0443 | -0.0270 | 0.0827 |
|  |  |  |  | (0.186) | (0.186) | (0.188) |
| Labor earnings (ln) |  |  |  | 0.000661 | -0.00213 | -0.00990 |
|  |  |  |  | (0.0206) | (0.0207) | (0.0213) |
| ISCED-97: 0 |  |  |  | ref. | ref. | ref. |
| ISCED-97: 1 |  |  |  | 0.433 | 0.491 | 0.594 |
|  |  |  |  | (0.941) | (0.943) | (0.945) |
| ISCED-97: 2 |  |  |  | 1.124 | 1.191 | 1.109 |
|  |  |  |  | (0.929) | (0.929) | (0.931) |
| ISCED-97: 3 |  |  |  | 1.209 | 1.212 | 1.130 |
|  |  |  |  | (0.918) | (0.918) | (0.919) |
| ISCED-97: 4 |  |  |  | 0.797 | 0.806 | 0.926 |
|  |  |  |  | (0.952) | (0.952) | (0.953) |
| ISCED-97: 5 |  |  |  | 0.994 | 1.013 | 0.978 |
|  |  |  |  | (0.920) | (0.922) | (0.923) |
| ISCED-97: 6 |  |  |  | 1.273 | 1.256 | 1.131 |
|  |  |  |  | (0.928) | (0.928) | (0.928) |
| Year FE | NO | YES | YES | NO | YES | YES |
| Survey FE | NO | NO | YES | NO | NO | YES |
| Country FE | NO | NO | YES | NO | NO | YES |
| Constant | -1.504*** | -2.696*** | -2.876*** | -1.850** | -3.365*** | -3.550*** |
|  | (0.0988) | (0.516) | (0.862) | (0.919) | (1.051) | (1.256) |
| R-squared | 0.000 | 0.002 | 0.003 | 0.001 | 0.003 | 0.004 |

N=127,999 (55,609 persons). Robust standard errors in parentheses. *** p<0.01, ** p<0.05, * p<0.1

***Table 8*** *Fixed effects quantile regressions on deciles of self-rated health*

|  | (1) | (2) | (3) | (4) | (5) | (6) | (7) | (8) | (9) |
| --- | --- | --- | --- | --- | --- | --- | --- | --- | --- |
|  | 0.1 | 0.2 | 0.3 | 0.4 | 0.5 | 0.6 | 0.7 | 0.8 | 0.9 |
| Volunteer | 0.881^***^ | 0.813^***^ | 0.779^****^ | 0.723^***^ | 0.652^***^ | 0.583^***^ | 0.541^**^ | 0.513^**^ | 0.453 |
|  | (0.310) | (0.248) | (0.221) | (0.187) | (0.169) | (0.188) | (0.214) | (0.235) | (0.287) |
| Age | -0.741 | -0.813 | -0.848 | -0.906 | -0.981 | -1.052 | -1.097 | -1.126 | -1.189 |
|  | (2.602) | (2.080) | (1.854) | (1.565) | (1.419) | (1.578) | (1.794) | (1.970) | (2.409) |
| Married | 0.540 | 0.442 | 0.392 | 0.313 | 0.210 | 0.111 | 0.0496 | 0.00951 | -0.0772 |
|  | (0.475) | (0.380) | (0.339) | (0.286) | (0.259) | (0.288) | (0.328) | (0.360) | (0.440) |
| Paid job | 2.173^***^ | 1.918^***^ | 1.791^***^ | 1.585^***^ | 1.319^***^ | 1.063^***^ | 0.905^***^ | 0.801^**^ | 0.577 |
|  | (0.456) | (0.365) | (0.325) | (0.274) | (0.249) | (0.276) | (0.314) | (0.345) | (0.422) |
| Retired | 2.618^***^ | 2.427^***^ | 2.332^***^ | 2.178^***^ | 1.978^***^ | 1.787^***^ | 1.668^***^ | 1.591^***^ | 1.423^***^ |
|  | (0.561) | (0.449) | (0.400) | (0.338) | (0.306) | (0.340) | (0.387) | (0.425) | (0.520) |
| Labor earnings | 0.0081 | 0.0170 | 0.0214 | 0.0286 | 0.0378 | 0.0467 | 0.0522 | 0.0558 | 0.0636 |
|  | (0.0500) | (0.0399) | (0.0356) | (0.0300) | (0.0272) | (0.0303) | (0.0345) | (0.0378) | (0.0463) |
| ISCED-97: 0 | ref. | ref. | ref. | ref. | ref. | ref. | ref. | ref. | ref. |
| ISCED-97: 1 | -2.791 | -2.757^*^ | -2.740^*^ | -2.712^**^ | -2.676^**^ | -2.641^**^ | -2.620^*^ | -2.605 | -2.575 |
|  | (2.092) | (1.673) | (1.491) | (1.258) | (1.141) | (1.269) | (1.443) | (1.584) | (1.937) |
| ISCED-97: 2 | -0.0579 | -0.244 | -0.336 | -0.486 | -0.680 | -0.866 | -0.982 | -1.057 | -1.220 |
|  | (1.870) | (1.495) | (1.332) | (1.124) | (1.020) | (1.134) | (1.290) | (1.416) | (1.731) |
| ISCED-97: 3 | 0.564 | 0.266 | 0.118 | -0.124 | -0.435 | -0.733 | -0.918 | -1.039 | -1.301 |
|  | (1.708) | (1.365) | (1.217) | (1.027) | (0.931) | (1.035) | (1.178) | (1.293) | (1.581) |
| ISCED-97: 4 | 1.120 | 0.813 | 0.660 | 0.411 | 0.0899 | -0.218 | -0.409 | -0.534 | -0.804 |
|  | (2.135) | (1.707) | (1.521) | (1.284) | (1.164) | (1.295) | (1.473) | (1.617) | (1.977) |
| ISCED-97: 5 | 1.523 | 1.091 | 0.877 | 0.528 | 0.0774 | -0.355 | -0.622 | -0.798 | -1.177 |
|  | (1.886) | (1.508) | (1.344) | (1.134) | (1.029) | (1.144) | (1.301) | (1.429) | (1.747) |
| ISCED-97: 6 | 1.103 | 0.843 | 0.714 | 0.504 | 0.232 | -0.0277 | -0.189 | -0.294 | -0.523 |
|  | (2.001) | (1.600) | (1.426) | (1.204) | (1.092) | (1.214) | (1.380) | (1.516) | (1.853) |

N=952026. Year dummies included. Standard errors in parentheses. *** p<0.01, ** p<0.05, * p<0.1

## APPENDIX B: ESTIMATES BY COUNTRY AND BY SURVEY

### Robustness checks: Different coefficients across countries, but the overall results are robust

To examine whether the results differ across countries and surveys, we provide robustness checks of all estimates for each country and each survey separately.

*Cross-sectional differences*. The cross-sectional bivariate health difference between volunteers and non-volunteers varies considerably between countries (see Figure C1 in the appendix). It is particularly large for respondents in Estonia (46%), Spain (27%), Luxembourg (26%), Hungary (25%) and Croatia (24%). These are all countries from the SHARE data, which is the survey with the largest relative health difference between non-volunteers and volunteers (26%). The health advantage tends to be larger in eastern Europe and smaller in the north-western countries. For four countries we have a particularly large number of observations. The health advantage in these countries seems to be somewhat smaller than in other countries: it is 7.5% in the UK, 4.7% in Switzerland, 5.3% in Germany, and 6.1% in the Netherlands.

*OLS models*. Figure C2 in the appendix shows the volunteering coefficients of OLS regression models, controlling for individual covariates and including dummies for year and (if applicable) survey or country. The OLS regression coefficient is negative and non-significant for Poland; positive and non-significant for Hungary, Portugal, Ireland, Greece and Croatia; and positive and significant for the remaining countries. Across all surveys, too, the OLS regression coefficients are significant. We find the largest coefficient again in the SHARE, but the coefficient of 5.1 points is much smaller than the cross-sectional mean difference (Figure C1) due to the control variables.

*Fixed-effects models*. When adding fixed effects for individual respondents, more subsample coefficients become non-significant (Figure C3). Portugal seems an outlier with a relatively large, yet non-significant, coefficient (-3.74). Germany, Switzerland and the UK have the largest number of respondents and provide the most precise estimates, which are robustly positive (0.42, 0.78 and 1.09, respectively). Rerunning the model excluding one of these countries does not change the conclusion from the main specification.

*First-difference models*. Figure C4 shows the results of first-difference models by country and by survey. The coefficient of starting volunteering (vs. remaining inactive) is negative and significant in Slovenia; positive and significant in Switzerland, the UK, Belgium and Spain; and not significant in the other countries. The coefficient is non-significant too in Germany, the country with the largest subsample. Most of the coefficients are positive, and the confidence intervals are relatively large due to the smaller subsamples. Rerunning the model while excluding an influential country like the United Kingdom does not change the overall results.

We find similar results for the model of continued volunteering (vs. leaving voluntary work). The volunteering coefficient is negative and significant in Israel, while it is positive and significant in Switzerland, the UK and Sweden. In the largest subsample, Germany, the coefficient is not significant. There is not one country or survey that on itself is responsible for the overall positive association.

*Fixed effects quantile regressions*. We run fixed effects quantile regressions separately for four single-country surveys with at least 10 years’ data, including GSOEP (n=328,093), BHPS (n=209,967), SHP (n=137,647), and LISS (n=34219). Because the bias of the estimator increases as T decreases (Machado and Santos Silva 2019), we did not run fixed effects quantile regressions for individual surveys or countries with fewer years of data as we did with other robustness checks. Nonetheless, the four surveys we discuss below represent about 75 percent of the whole sample.

For GSOEP (Germany), BHPS (UK), and SHP (Switzerland), we find patterns that are similar to the whole sample analysis, with the positive association between volunteering and health decreasing from the lowest to highest decile of health. However, the association is statistically significant only from the third to the sixth decile in GSOEP and SHP, and from the median to the top decile in BHPS. Compared to the whole sample results, the magnitude of the association is smaller for Germany at every decile (from .668 to .184), but larger for the UK sample (from 1.280 to .904). On the other hand, the results using the LISS (the Netherlands) are not significant at any decile.

When combining data from SHARE, the quantile regression results for Germany and Switzerland become non-significant, although the coefficient on volunteering still decreases from the lowest to the highest decile of health. Results are still non-significant for all deciles for the Netherlands with combined data from LISS, GINPS, and SHARE. Overall, with the exception of the Netherlands, we find differential associations between volunteering and health across health distributions by individual surveys, but the magnitude and statistical significance of the association may vary by survey.

***Fig. 5*** *Average health among volunteers and non-volunteers, by country and by survey*

**
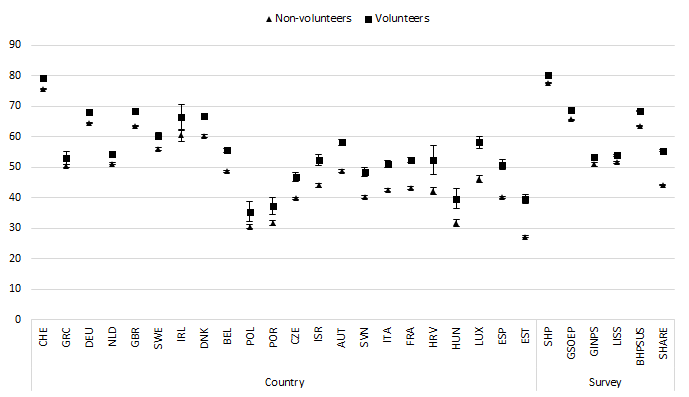
**

***Fig. 6*** *Coefficients of volunteering on self-rated health from OLS regression models with full controls, by country and by survey*

**
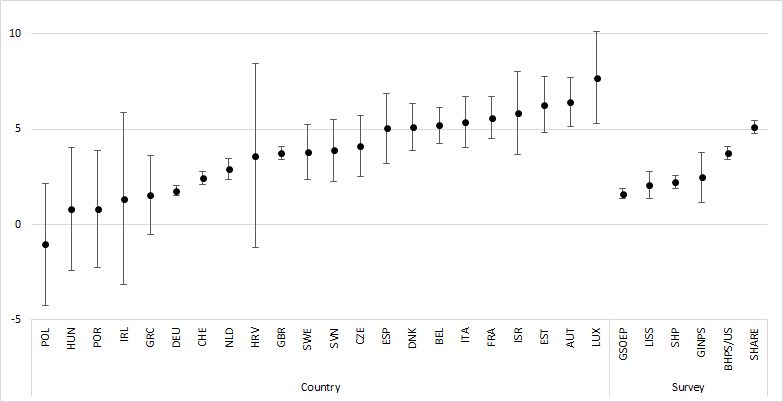
**

*Figure C3: Coefficients of volunteering on self-rated health from regression models with fixed effects in individuals and full controls, by country and by survey*

**
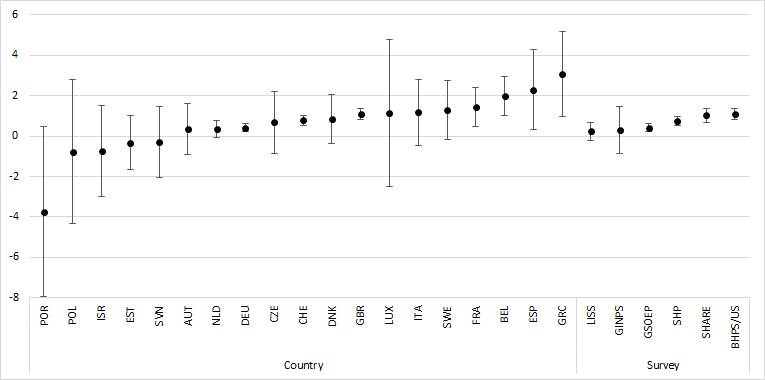
**

***Fig. 7***

*(a) Coefficients of starting volunteering (vs. not volunteering) on self-rated health in first-difference regression models with full controls, by country and by survey*

*
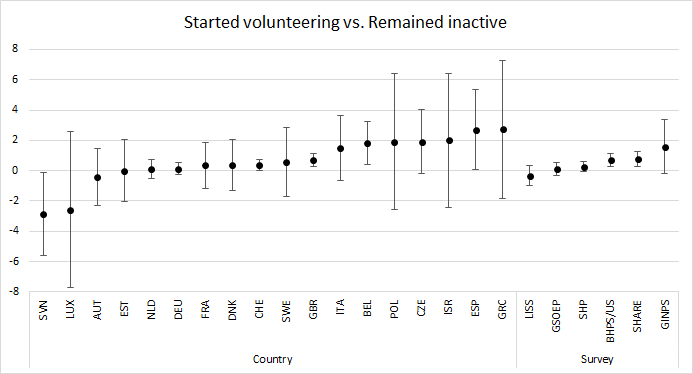
*

*(b) Coefficients of continued volunteering (vs. quitting volunteering) on self-rated health in first-difference regression models with full controls, by country and by survey*

*
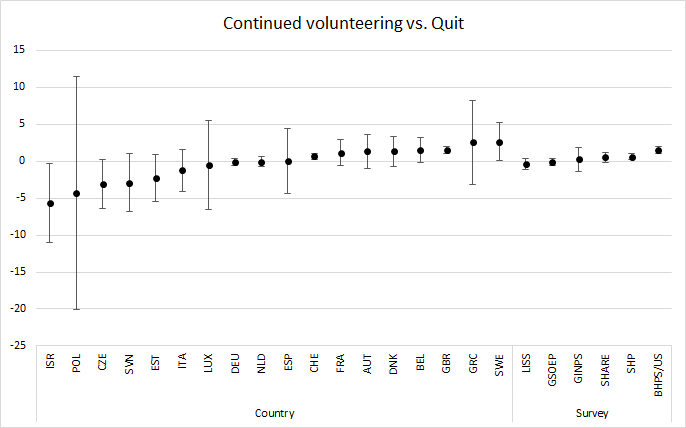
*

## APPENDIX D: EXPLORATORY ANALYSES USING DYNAMIC PANEL DATA ESTIMATORS

Following our pre-analysis plan, we have considered using a dynamic panel data (DPD) estimator (Equation (1)). It allows lagged health to affect volunteering and also includes the time-dependence of health, that is, allowing the dependent variable to depend on its values from prior time periods (in a way that is not explained by other regressors). Because including the dependent variable on the right-hand side of the equation biases standard panel estimators, valid instruments are needed to obtain unbiased consistent estimates. However, our exploratory analyses did not find valid instruments for Equation (2) that are supported by diagnostic test results.


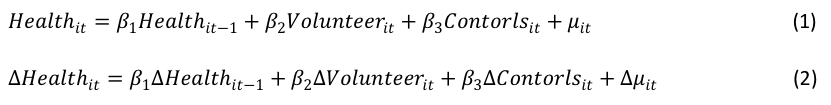


We conducted exploratory analysis in selected surveys (e.g. SHP) because estimating DPD models is computationally intensive (which uses iteration). An important aspect of the DPD estimator is using an individual’s history within the panel as instruments for explanatory variables. Specifically, we experimented with both difference generalized methods of moments estimators where differences were instrumented by levels (difference GMM; Arellano and Bond, 1991), as well as system generalized methods of moments estimators where lagged differences were used as instruments for the level model and lagged levels as instruments for the first-difference model (System GMM; Blundell & Bond, 1998). To be valid instruments, the lagged dependent and explanatory variables must provide an exogenous source of variation for current volunteering. Unfortunately, we did not find a valid model that passed the Hansen J test of over-identification for instrument validity.

Moreover, the DPD model assumes that one’s decision to volunteer or not is based on some expectations of health (i.e. the evaluation of past health). While this might be plausible for a small group of individuals who strategically choose to volunteer in order to maintain their health, it may not be representative of the majority. In contrast, it would be more reasonable to assume that the contemporaneous health status, rather than lagged health measured in the prior wave (e.g. one, two, or even four years ago), is a relevant factor in decisions to volunteer or not. Indeed, using a fixed effect logit model with current volunteering as dependent variable and lagged health and controls as regressors, we found a very small association between lagged health and current volunteering (b= .0018, p<.001).

Finally, the DPD estimator looks for historical values of health beyond certain lags to be potentially valid instruments since they will be exogenous to current health. Based on the second order autocorrelation test, we found that it generally needed adjusting for two to three lags of health measures. However, it is plausible that self-rated health reflects persistent individual characteristics as well as long-run health behavior and investments in which the health status many years ago might not be strictly exogenous. Therefore, we conclude that DPD is not an appropriate model for our question.
